# Gut Microbiota and Related Electronic Multisensorial System Changes in Subjects With Symptomatic Uncomplicated Diverticular Disease Undergoing Rifaximin Therapy

**DOI:** 10.3389/fmed.2021.655474

**Published:** 2021-07-19

**Authors:** Antonio De Vincentis, Marco Santonico, Federica Del Chierico, Annamaria Altomare, Benedetta Marigliano, Alice Laudisio, Sofia Reddel, Simone Grasso, Alessandro Zompanti, Giorgio Pennazza, Lorenza Putignani, Michele Pier Luca Guarino, Michele Cicala, Raffaele Antonelli Incalzi

**Affiliations:** ^1^Unit of Internal Medicine, University Campus Bio Medico of Rome, Rome, Italy; ^2^Unit of Electronics for Sensor Systems, Department of Science and Technology for Humans and the Environment, University Campus Bio-Medico di Roma, Rome, Italy; ^3^Multimodal Laboratory Medicine Research Area, Unit of Human Microbiome, Bambino Gesù Children's Hospital, Istituto di Ricovero e Cura a Carattere Scientifico, Rome, Italy; ^4^Unit of Gastroenterology, University Campus Bio Medico of Rome, Rome, Italy; ^5^Emergency Department, Azienda Ospedaliera San Camillo Fornanini, Rome, Italy; ^6^Unit of Geriatrics, University Campus Bio Medico of Rome, Rome, Italy; ^7^Unit of Electronics for Sensor Systems, Department of Engineering, University Campus Bio-Medico di Roma, Rome, Italy; ^8^Department of Diagnostic and Laboratory Medicine, Unit of Microbiology and Diagnostic Immunology, Unit of Parasitology and Multimodal Laboratory Medicine Research Area, Unit of Human Microbiome, Bambino Gesù Children's Hospital, Istituto di Ricovero e Cura a Carattere Scientifico, Rome, Italy

**Keywords:** microbiota, e-tongue, e-nose, diverticular disease, rifaximin

## Abstract

**Background:** Intestinal dysbiosis might play a pathogenetic role in subjects with symptomatic uncomplicated diverticular disease (SUDD), but the effect of rifaximin therapy has been scantly explored with regard to gut microbiota variations in patients with SUDD.

**Aims:** To verify to which extent rifaximin treatment affects the gut microbiota and whether an electronic multisensorial assessment of stools and breath has the potential for detecting these changes.

**Methods:** Breath and stool samples were collected from consecutive patients with SUDD before and after a 7 days' therapy with rifaximin. Stool microbiota was assessed, and the electronic multisensorial assessment was carried out by means of the BIONOTE electronic (e-)tongue in stools and (e-)nose in breath.

**Results:** Forty-three subjects (female 60%, median age 66 years) were included, and 20 (47%) reported clinical improvement after rifaximin therapy. Alpha and beta diversity of stool microbiota did not significantly change after treatment, while a significant variation of selected taxa was shown (i.e., *Citrobacter, Coprococcus, Anaerotruncus, Blautia, Eggerthella lenta, Dehalobacterium, SMB53*, and *Haemophilus parainfluenzae*). Overall, the electronic multisensorial system suboptimally mirrored microbiota changes, but it was able to efficiently predict patients' clinical improvement after rifaximin with accuracies ranging from 0.81 to 0.98.

**Conclusions:** In patients with SUDD, rifaximin administration is associated with significant variation of selected taxa. While inaccurate in predicting gut microbiota change, an electronic multisensorial system, made up of e-tongue and e-nose, was able to predict clinical improvement, thus potentially qualifying as an easy and cheap tool to forecast subjects taking most likely benefit from rifaximin therapy.

## Introduction

Colonic diverticulosis is a complex multifactorial disorder, in which dysbiosis could play a key role. It affects up to one third of people over the age of 60 years and causes symptoms including abdominal pain or bloating and changes in bowel habit (condition termed symptomatic uncomplicated diverticular disease—SUDD) in 20% of cases (1). Approximately 10–25% of patients with SUDD may develop acute diverticulitis (2). The fact that the small bowel diverticula are associated with bacterial overgrowth and most complications of colonic diverticular disease are bacterial in nature and may benefit from antibiotic therapy or fecal stream diversion further supports the importance of gut microbiota in diverticular disease pathogenesis (3). Fiber deficiency, attributed to a Western diet, plays a crucial role, considering that DNA sequencing confirms that fecal microbiota composition is affected by consumption of supplemental fibers (4). A low-fiber diet not only affects colonic motility but can account for the microbiota composition bending toward a prevalence of *Bacteroides* (5). Low-grade inflammation, altered intestinal microbiota, visceral hypersensitivity, and an abnormal colonic motility are likely to play a variable pathogenetic role (6). The presence of inflammation and dysbiosis in SUDD validates the responsiveness to anti-inflammatory medications like non-absorbable enteral antibiotics such as rifaximin, a semisynthetic antibiotic synthesized in 1982 from rifamycin (7). Rifaximin has a broad spectrum of antibacterial action, is unlikely to induce bacterial resistance (7), and decreases the metabolic activity of the intestinal bacterial flora and the degradation of dietary fiber (8). Rifaximin acts by binding to the beta subunit of bacterial DNA-dependent RNA polymerase resulting in the inhibition of bacterial RNA synthesis (9). It has *in vitro* bactericidal and bacteriostatic activity against aerobic and anaerobic gram-positive and gram-negative species, being also able to reduce bacterial virulence and translocation and to inhibit bacterial adherence to gut mucosa (10). In fact, cyclic administration of rifaximin with dietary fiber supplementation outperforms simple dietary fiber supplementation in reducing both symptoms and complication frequency of SUDD (11). In conclusion, the better comprehension of the inflammatory patterns and the gut microbiota has increased the therapeutic options: current evidence enhances the therapeutic role of rifaximin (as well as mesalazine that acts directly on the gastrointestinal epithelium) in the treatment of SUDD symptoms, and the fiber supplementation is still recommended in SUDD by much of the international guidelines even if its use is not supported by the recent evidence. Unfortunately, the only available study of rifaximin effects on gut microbiota refers to only four women, being thus less than exploratory in nature (12). Thus, we purposed to verify to which extent rifaximin treatment affects the gut microbiota and whether an electronic multisensorial assessment of stools and breath has the potential for detecting these changes. Indeed, the genetic study of microbiota is cumbersome and requires both time and money. Instead, both a gas sensor array (e-nose) and the liquid sensor array (e-tongue) qualify as very simple and inexpensive methods for assessing, respectively, the spectrum of volatile organic compounds and the electrical impedance of a given liquid, which clearly reflect its physical properties and chemical composition (13). Both e-nose and e-tongue have displayed a wide spectrum of diagnostic and classificatory properties in different conditions, e.g., in liver diseases, chronic obstructive pulmonary disease, asthma, and selected tumors for the e-nose (14–19), in ascites, pleural effusion, urinary tract infections, and wound infections for e-tongue (20–23). Thus, e-nose and e-tongue might be able to catch the rifaximin-induced changes in gut microbiota, allowing either to interpret or to monitor the response to rifaximin in SUDD patients.

## Methods

### Study Setting and Participants

In this prospective longitudinal study, consecutive subjects with a diagnosis of SUDD were recruited at the Campus Bio-Medico University Hospital of Rome (Italy) from January to July 2017. Both design and size of this study are consistent with the intention of performing a proof-of-concept study testing the diagnostic properties of e-nose and e-tongue toward genetically proved changes in gut microbioma induced by rifaximin. SUDD was defined as the presence of abdominal pain, bloating, and/or bowel habit changes that include diarrhea, constipation, or a mixed bowel habit, in patients with diverticulosis in the absence of macroscopic inflammation and of any complications (stenosis, abscesses, fistula) (2, 24). Subjects referring allergy to rifaximin; or taking medication with a potential modifying role on microbiota (i.e., antibiotics, prebiotics, probiotics, proton pump inhibitors) in the previous month; or with active cancer, COPD exacerbation, end stage of liver, or kidney disease; or suffering from inflammatory bowel disease were excluded. Finally, considering the well-known influence of diet on intestinal microbiota, only subjects following a Mediterranean normocaloric omnivorous diet (2,000–2,100 and 2,500–2,800 kcal/diet for women and men, respectively) were included.

Since a reduction of potentially pathogenic components of the intestinal microbiota was expected after rifaximin therapy in at least the 70% of participants, the enrollment of 20 subjects was considered adequate to guarantee a statistical power of 80% assuming an alpha error of 0.05. Based on previous experience with multisensorial systems (14, 15, 20–23), this sample size was increased to at least 40 subjects in order to allow the investigation of the discriminative and classificatory capacities of these systems toward rifaximin-induced microbiome changes.

All the main socio-demographic and clinical variables along with blood tests were collected for each participant. Nutritional assessment was performed through the Mini Nutritional Assessment (MNA) (25), abdominal symptoms (i.e., pain and bloating) were scored as mild, moderate, or severe, and bowel habits were assessed through the Bristol Stool Scale. Eligible subjects were evaluated at baseline and, then, were prescribed with a cycle of 7 days of rifaximin 800 mg/diet. A follow-up evaluation was performed after the completion of the antibiotic therapy. Breath and fecal samples were collected for each participant at baseline and at follow-up for the multisensorial system analysis and for the gut microbiota analysis. The study protocol was approved by the local Ethical Committee (n 47/2016), and all participants signed an informed consent.

### Gut Microbiota Analysis

Fecal samples were collected by each subject in the same morning of the outpatient visit. DNA was extracted from 200 mg of stools using the QIAamp Fast DNA Stool Mini Kit (Qiagen, Hilden, Germany), following the manufacturer's instructions. The 16S rRNA V3–V4 variable region (~460 bp) was amplified by using the primer pairs described in the MiSeq rRNA Amplicon Sequencing protocol (Illumina, San Diego, CA, USA). The PCR reactions were set up using a 2 × KAPA HiFi HotStart ReadyMix (KAPA Biosystems Inc., Wilmington, MA, USA) following the manufacturer's protocol. AMPure XP beads (Beckman Coulter Inc., Beverly, MA, USA) were employed to clean DNA amplicons from primers and dimer primers. A unique combination of Illumina Nextera adaptor-primers for each sample was incorporated in amplicons by a second amplification step. The final library was cleaned up and quantified using Quant-iT™ PicoGreen® dsDNA Assay Kit (Thermo Fisher Scientific, Waltham, MA, USA). Samples were pooled together before the sequencing on an Illumina MiSeq™ platform according to the manufacturer's specifications to generate paired-end reads of 300 base-length. QIIME v1.9 software (26) was used to filter raw reads for quality, read length, and chimera presence. Cleaned sequences were then clustered into operational taxonomic units (OTUs) with a 97% clustering threshold of pairwise identity. OTUs' representative sequences were aligned using PyNAST v.0.1. software (27) against the Greengenes 13_08 database with a 97% similarity for bacterial sequences. All raw sequences have been archived in the NCBI database: PRJNA731467 (https://www.ncbi.nlm.nih.gov/bioproject).

### Multisensorial System Analysis on Breath and Fecal Samples

The multisensorial system was made up of the BIONOTE e-Tongue for liquids and of the BIONOTE e-Nose for gases (13). Breath collection was performed in the morning by means of the Pneumopipe® (European Patent n 12425057.2), with all participants who have been fasting and smoke-free for at least 8 h. The breath samples were stored in adsorbent cartridges (Tenax GR by Supelco) at −20°C after collection. Before analysis, the VOC mixture was desorbed with a thermal procedure at four different temperatures (50, 100, 150, and 200°C) and, then, analyzed by the BIONOTE e-Nose (13). This instrument is composed of eight transducers consisting of quartz crystals with a resonance frequency of 20 MHz in thickness shear mode, functionalized with a combination of anthocyanins extracted from three different plant tissues: red rose, red cabbage, and blue hortensia. Each VOC in the mixture binds to the different anthocyanins in the measure cell, thus causing a frequency shift of the respective quartz forming the reference value: this frequency shift is acquired by the system as the sensor response. The final dataset is composed of a fingerprint of 32 responses for each sample, which derive from the registration of the eight-sensor behavior at the four different temperatures of VOC desorption from the cartridge.

Fecal samples were collected by each subject in the same morning of the outpatient visit and stored at +4°C. Before the analysis, feces were prepared for the analysis by diluting 50 mg in 5 ml of distilled water and subsequently centrifuged at 10°C at 1,000 relative centrifugal forces for 10 min. The supernatant was taken with a pipette and analyzed by the BIONOTE e-Tongue (13). This instrument consists of voltammetric screen-printed electrodes controlled by a high-stability electronic interface. The sensor probe is made up of three electrodes: a silver working, a platinum reference, and a gold counter electrode. The applied input signal consists of a triangular waveform with a working range from −1 to 1 V with 500 input voltages and 500 corresponding output current values. The time duration of the complete measurement process is of about 90 s, repeated five times for reproducibility assessment. Data array is made up by the characteristic fingerprints extracted by each voltammogram registered for the measured samples. The setup of the parameters for the acquisition, the number of samples, and the sampling interval are controlled by a dedicated software interface.

### Analytical Approach

Data were presented by means of descriptive statistics and compared using non-parametric tests. Alpha diversity of the gut microbiota was measured on the raw data by the Good's coverage, Chao-1, and Shannon indices, using the Wilcoxon signed-rank test to assess differences after rifaximin therapy. For further analyses, OTUs not seen in at least 20% of the samples or with a relative abundance <1% in the total dataset were removed. Principal component analysis (PCoA) on unweighted and weighted UniFrac distance matrices was used as ordination method to compare gut microbiota of subjects pre- and post-rifaximin therapy by permutational multivariate analysis of variance (PERMANOVA). In addition, the differential abundance analysis of gut bacteria was conducted through the negative binomial distribution on raw counts normalized by “size factors,” taking into account the sequencing depth between the samples. The differences in bacterial abundance were expressed as log_2_ fold change (log_2_FC). Analyses of gut microbiota changes were stratified by gender and by presence of abdominal pain, and linear mixed models were applied to verify the related impact in the amount of alpha diversity change after rifaximin therapy.

The ability of e-tongue and e-nose to predict gut microbiota changes or clinical improvement after rifaximin therapy was verified using partial-least-squared discriminant analysis (PLS-DA) with 10-fold cross-validation. Predictive capacities were expressed with the root-mean-square-error cross validation (RMSECV) to aggregate in a single measure of predictive power the magnitudes of the machine errors in prediction of continuous variables (e.g., Shannon and Chao-1 indices). Since RMSECV expresses the prediction error in the same unit of the original measurement, it cannot be compared between different variables. To allow comparability, RMSECV% was computed standardizing RMSECV by the 95% interval of the variable-specific distribution. Conversely, for dichotomous outcomes (i.e., clinical improvement), overall accuracy, sensitivity, specificity, and positive (PPV) and negative (NPV) predictive values were computed. All the analyses were performed using R version 4.0.2 (The R Foundation for Statistical Computing, Vienna, Austria).

## Results

### Pre–post Rifaximin Clinical and Biochemical Characteristics

Forty-three subjects with SUDD were included in the study (Table 1). The median age was 66 years, and 26 (60%) were women, with a median BMI of 24.5 kg/m^2^. Sixteen subjects (37%) were at risk of malnutrition, and no malnourished subjects were identified. At baseline, all subjects complained about abdominal bloating [mild: 29 (67%); moderate: 4 (9.3%); and severe: 10 (23%)] and 18 (42%) referred also abdominal pain [mild: 13 (72.2%); moderate: 5 (27.8%)]. According to the Bristol Stool Scale, 12 participants (28%) had diarrheal stool, while only 2 (4.7%) were constipated.

**Table 1:** Main clinical and biochemical characteristics of the study cohort at baseline and after rifaximin therapy (follow-up).

**Characteristic**	***N*** **= 43**		
Age (years)	66 (61, 73)		
Sex (female)	26 (60%)		
BMI (kg/m^2^)	24.5 (22.6, 27.4)		
Malnutrition (MNA)		
*Well-nourished*	27 (63%)		
*At risk of malnutrition*	16 (37%)		
*Malnourished*	0 (0%)		
DICA classification		
1	40 (93%)		
2	3 (7%)		
3	0 (0%)		
Clinical improvement after rifaximin^*^	20 (47%)		
	**Baseline**	Follow-up^**^	***p***
Hemoglobin (g/dl)	14.1 (12.8, 14.9)	14.1 (13.2, 14.9)	0.357
Leukocytes (/mm^3^)	6,000 (5,110, 7,165)	6,050 (4,930, 7,205)	0.947
Lymphocytes (/mm^3^)	1,970 (1,560, 2,315)	1,990 (1,500, 2,425)	0.476
C-reactive protein (mg/dl)	1.4 (0.5, 3.7)	0.5 (0.5, 3.0)	0.194
ESR (mm/h)	36 (26, 42)	33 (20, 43)	0.101
Bristol Stool Scale (linear)	5 (4, 6)	4 (3, 5)	<0.001
Bristol Stool Scale (categories)			0.011
*1–2 (constipation)*	2 (4.7%)	6 (14%)	
*3–5 (normal)*	29 (67%)	35 (81%)	
*6–7 (diarrhea)*	12 (28%)	2 (4.7%)	
Abdominal pain (presence)	18 (42%)	10 (23%)	0.05
Abdominal pain (severity)			0.05
*Absence*	25 (58%)	33 (77%)	
*Mild*	13 (30%)	10 (23%)	
*Moderate*	5 (12%)	0 (0%)	
*Severe*	0 (0%)	0 (0%)	
Abdominal bloating (presence)	43 (100%)	43 (100%)	1
Abdominal bloating (severity)			0.003
*Absence*	0 (0%)	0 (0%)	
*Mild*	29 (67%)	42 (98%)	
*Moderate*	4 (9.3%)	1 (2.3%)	
*Severe*	10 (23%)	0 (0%)	

*Continuous variables are expressed as median with interquartile range, while categorical variables are displayed as numbers with percentages. p-values are from the Wilcoxon signed-rank test for continuous variables or from McNemar's or marginal homogeneity test for categorical variables*.

* *Clinical improvement defined as relief of abdominal pain or any improvement of abdominal bloating or normalization of bowel habits with a Bristol Stool Scale 3–5*.

** *After a 1-week course of rifaximin 800 mg/die*.

*MNA, Mini Nutritional Assessment; BMI, body mass index; ESR, erythrocyte sedimentation rate; DICA classification, “Diverticular Inflammation and Complication Assessment” classification*.

After rifaximin therapy, no significant differences were observed regarding the main inflammatory markers (leukocytes, C-reactive protein, and erythrocyte sedimentation rate). Conversely, patients reported a significant improvement of symptoms in terms of abdominal pain (*p* = 0.05), bloating (*p* = 0.003), and bowel habits (*p* = 0.011—Table 1). Overall, 20 subjects (47%) experienced a clinical improvement after therapy, defined as relief of abdominal pain or any improvement of abdominal bloating or normalization of bowel habits.

### Influence of Rifaximin Therapy on Gut Microbiota

**Figure 1:**
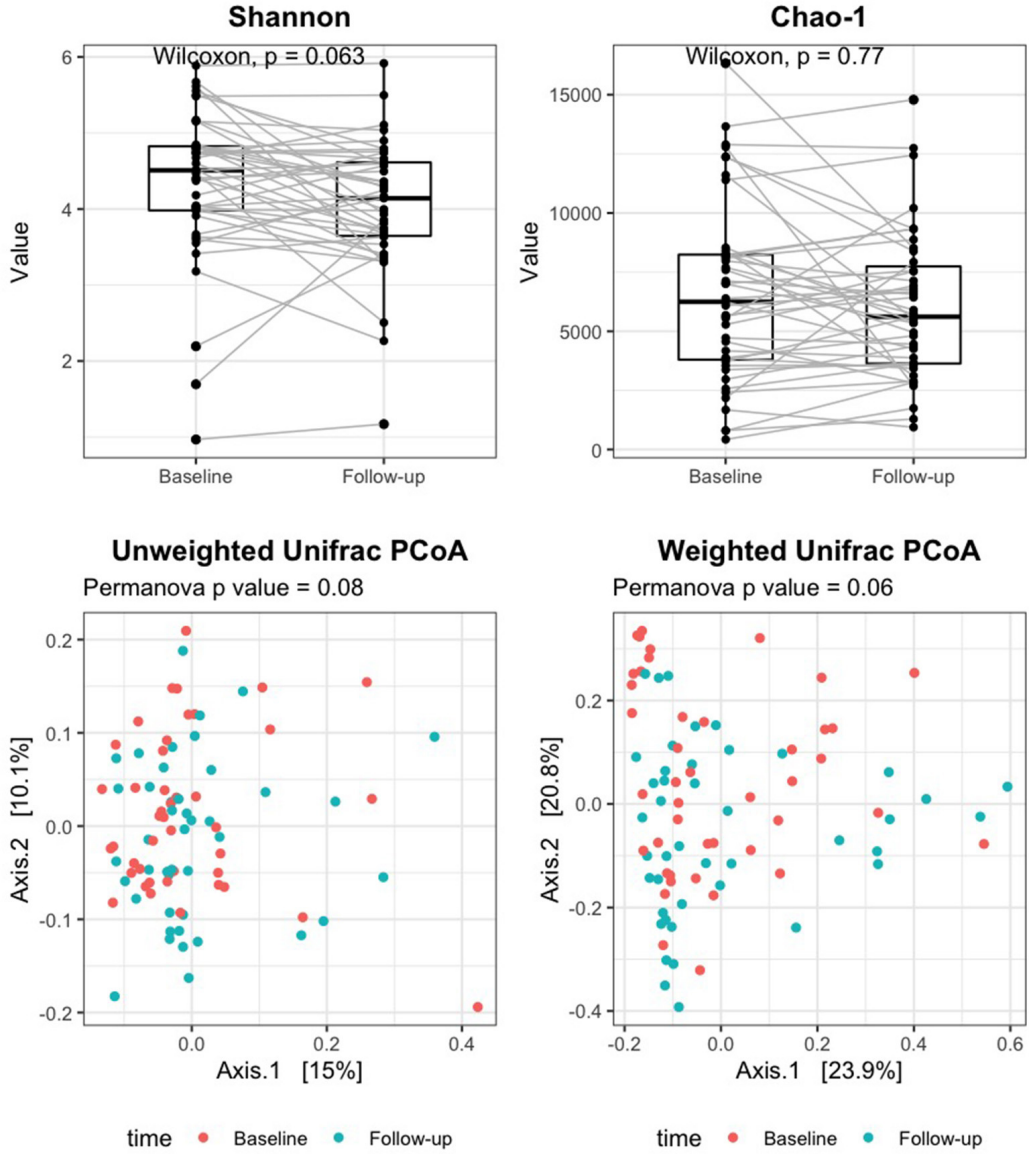
Change of alpha and beta diversity measures after rifaximin therapy in SUDD patients. Alpha diversity of the gut microbiota was measured on the raw data by the Shannon and Chao-1 indices (upper panels). Beta diversity was assessed by principal component analysis (PCoA) on unweighted and weighted UniFrac distance matrices (lower panels). The Wilcoxon signed-rank test and permutational multivariate analysis of variance (PERMANOVA) were applied to assess differences in alpha and beta diversity measures after rifaximin therapy, respectively.

Good's coverage averaged 96% in baseline samples and 97% in follow-up, indicating that most of the OTUs in the samples were detected. Alpha diversity measures did not significantly change after rifaximin therapy [Shannon index: baseline 4.5 (4.0–4.8) vs. follow-up 4.1 (3.6–4.6), *p* 0.06; Chao-1 index: baseline 6,246.1 (3,796.5–8,239.8) vs. follow-up 5,615.7 (3,634.5–7,741), *p* 0.77; Figure 1, upper panels]. Similarly, the UniFrac PCoA plot did not show a shift in the overall gut microbiota composition from baseline to post-rifaximin treatment (PERMANOVA *p* 0.08 for unweighted and 0.06 for weighted analysis; Figure 1, lower panels). Similar findings were observed after stratification according to gender and to the presence of abdominal pain (Supplementary Figure 1), and no difference was found in the amount of alpha diversity change after rifaximin therapy across gender (p for linear mixed model 0.40 and 0.92 for Shannon and Chao-1 indices, respectively) and across patients with/out abdominal pain (p for linear mixed model 0.26 and 0.42 for Shannon and Chao-1 indices, respectively).

**Figure 2:**
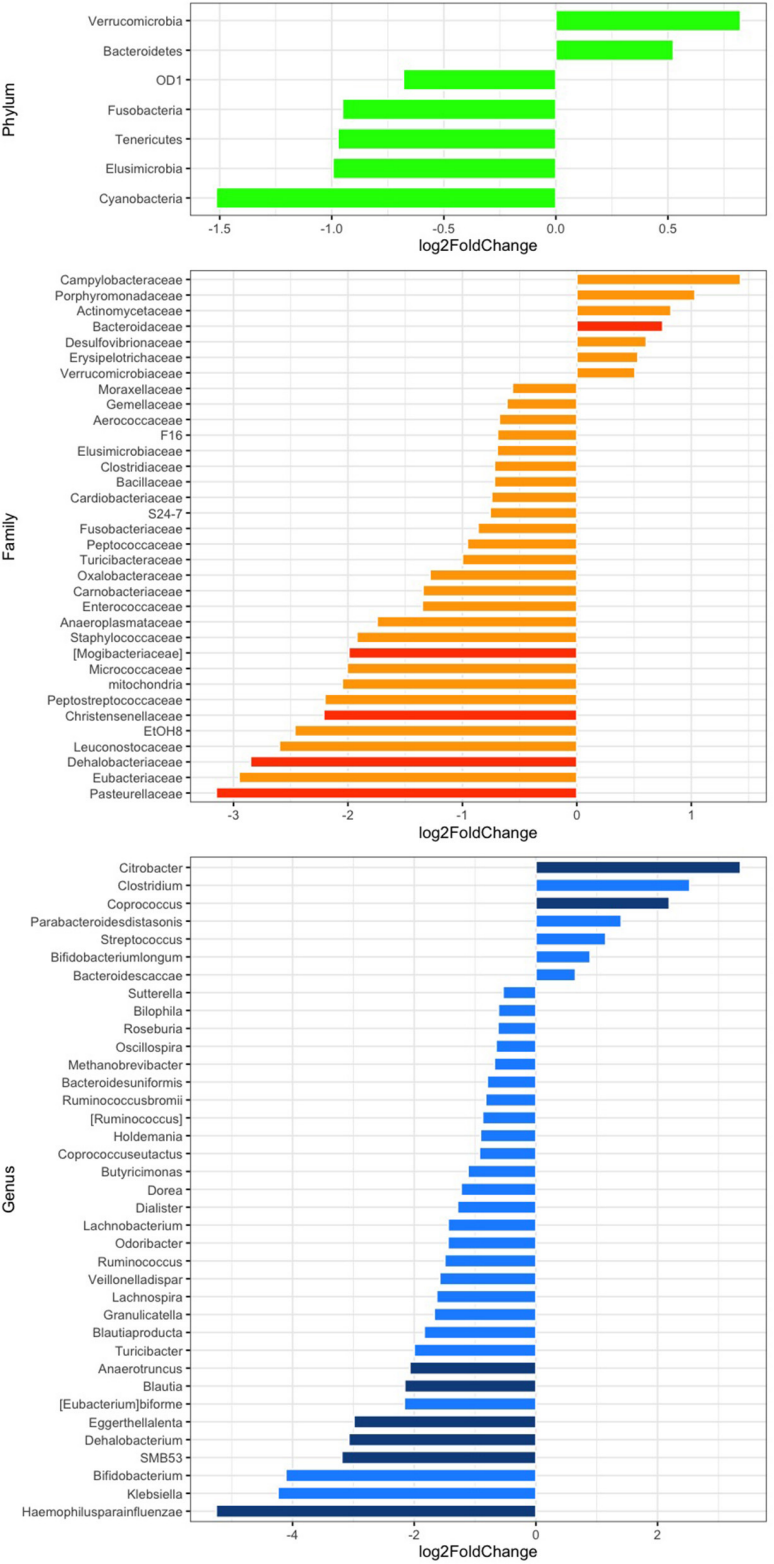
Differential abundance analysis of the gut microbiota composition at the phylum (green), family (orange), and genus (blue) levels after rifaximin therapy. Differential bacterial abundance is expressed as log2 fold change (log2FC); positive or negative values indicate an increase or decrease proportional to the absolute value of log2FC. Comparisons with a log2FC higher or lower than 0.5 are displayed. *p* < 0.05 adjusted for multiple comparisons with the Benjamini-Hochberg method (*p*-adj) are considered significant and represented by a darker color.

The relative abundance analysis revealed significant changes in selected families and genera (Figure 2). In particular, after rifaximin therapy the gut microbiota was enriched in Bacteroidaceae, *Citrobacter*, and *Coprococcus* and deficient in Mogibacteriaceae, Christensenellaceae, Dehalobacteriaceae, Pasteurellaceae, *Anaerotruncus, Blautia, Eggerthella lenta, Dehalobacterium, SMB53*, and *Haemophilus parainfluenzae* (*p*-adj <0.05) at the family and genus levels, respectively (Figure 2). Other selected families and genera showed large log_2_FC (>2 or <-2), but without reaching statistical significance: Peptostreptococcaceae, EtOH8, Leuconostocaceae, Eubacteriaceae, *Clostridium, Bifidobacterium*, and *Klebsiella* (Figure 2). Subgroup analyses revealed a significant decrease of Pasteurellaceae, Clostridiaceae, *Blautia, Veillonella dispar*, and *Haemophilus parainfluenzae* in men and of Christensenellaceae in women. *Parabacteroides* were significantly increased in men (Supplementary Figures 2A,B). Selected variations at the phylum, family, and genus levels according to the presence of abdominal pain have been also evidenced and are reported in Supplementary Figures 2C,D.

### Electronic Multisensorial System Prediction of Gut Microbiota and of Clinical Outcomes

Predictive performances of the electronic multisensorial system toward alpha diversity measures of gut microbiota in pre–post rifaximin samples are reported in Table 2. E-tongue and e-nose predicted Shannon index and Shannon index variation after rifaximin with a RMSECV% between 20 and 25 (RMSECV 0.62–0.95). A similar range of RMSECV% was observed for the prediction of Chao-1 index and Chao-1 index variation after rifaximin (19–26) with RMSECV between 2,292 and 3,314. The integration of e-tongue and e-nose data only minimally improved predictive performances (Table 2). The abilities of both e-tongue and e-nose to predict the main phyla, families, and genera variation were generally suboptimal, with exception made for a few selected cases (Supplementary Table 1).

**Table 2:** E-tongue and e-nose prediction of alpha measure variation in patients with SUDD undergoing rifaximin therapy.

	**E-tongue**		**E-nose**		**E-tongue + E-nose**	
	**RMSECV**	RMSECV %^*^	**RMSECV**	RMSECV %^*^	**RMSECV**	RMSECV %^*^
Prediction of Shannon index	0.88	22	0.95	24	0.80	20
Prediction of Shannon index change after rifaximin	0.62	20	0.78	25	0.50	16
Prediction of Chao-1 index	3,226	25	3,314	26	3,222	25
Prediction of Chao-1 index change after rifaximin	2,292	19	2,844	23	2,356	20

* *RMSECV% represents the ratio between the root mean squared error in cross validation (RMSECV) and the 95% interval of the distribution of each index/index variation*.

Conversely, the accuracy for the discrimination of pre/post rifaximin samples was 0.81 (0.72–0.89) for e-tongue, 0.9 (0.81–0.95) for e-nose, and 0.87 (0.78–0.93) for the integration of both techniques (Table 3). Both e-tongue and e-nose evidenced good to excellent capacities to predict clinical outcome after rifaximin therapy. Two different potential predictors were tested to this purpose, i.e., the sensors' responses obtained from the analysis of only pre-rifaximin samples, or the change in sensors' responses obtained from the analysis of both pre- and post-rifaximin samples. Indeed, the analysis of only pre-rifaximin samples could correctly predict the occurrence of clinical improvement after treatment in 36/43 subjects with e-tongue [accuracy 0.84 (0.69–0.93), sensitivity 0.91, specificity 0.75, PPV 0.81, NPV 0.88] and in 41/43 subjects with e-nose [accuracy 0.95 (0.84–0.99), sensitivity 1.00, specificity 0.90, PPV 0.92, NPV 1.00]. The integration of both techniques did not lead to an improvement of the predictive performances [accuracy 0.81 (0.67–0.92), sensitivity 0.87, specificity 0.75, PPV 0.80, NPV 0.83]. Similarly, the change in sensors' responses after rifaximin could efficiently predict clinical improvement [accuracy 0.84 (0.69–0.93) for e-tongue; accuracy 0.81 (0.67–0.92) for e-nose]. In this case, the integration of both techniques could further increase the accuracy to 0.98 (0.88–1) (sensitivity 1.00, specificity 0.95, PPV 0.96, NPV 1.00).

**Table 3:** E-tongue and e-nose discrimination of pre/post-rifaximin sample and prediction of clinical improvement.

**Discrimination of pre/post-rifaximin samples**														
**E-tongue**					**E-nose**					**E-tongue + E-nose**				
		**Reference**					**Reference**					**Reference**	
		***Baseline***	***Follow-up***				***Baseline***	***Follow-up***				***Baseline***	***Follow-up***	
**Predicted**	*Baseline*	34	7		**Predicted**	*Baseline*	39	5		**Predicted**	*Baseline*	38	6	
	*Follow-up*	9	36			*Follow-up*	4	38			*Follow-up*	5	37	
**Accuracy**	**Sensitivity**	**Specificity**	**PPV**	**NPV**	**Accuracy**	**Sensitivity**	**Specificity**	**PPV**	**NPV**	**Accuracy**	**Sensitivity**	**Specificity**	**PPV**	**NPV**
0.81 (0.72–0.89)	0.79	0.84	0.83	0.8	*0.9 (0.81–0.95)*	*0.91*	*0.88*	*0.89*	*0.9*	0.87 (0.78–0.93)	0.88	0.86	0.86	0.88
Prediction of clinical improvement by the analysis of only pre-rifaximin samples^*^														
**E-tongue**					**E-nose**					**E-tongue + E-nose**				
		**Reference**					**Reference**					**Reference**	
		***No***	***Yes***				***No***	***Yes***				***No***	***Yes***	
**Predicted**	*No*	21	5		**Predicted**	*No*	23	2		**Predicted**	*No*	20	5	
	*Yes*	2	15			*Yes*	0	18			*Yes*	3	15	
**Accuracy**	**Sensitivity**	**Specificity**	**PPV**	**NPV**	**Accuracy**	**Sensitivity**	**Specificity**	**PPV**	**NPV**	**Accuracy**	**Sensitivity**	**Specificity**	**PPV**	**NPV**
0.84 (0.69–0.93)	0.91	0.75	0.81	0.88	*0.95 (0.84–0.99)*	*1*	*0.9*	*0.92*	*1*	0.81 (0.67–0.92)	0.87	0.75	0.8	0.83
Prediction of clinical improvement by the analysis of both pre- and post-rifaximin samples^*^														
**E-tongue**					**E-nose**					**E-tongue + E-nose**				
		**Reference**					**Reference**					**Reference**	
		***No***	***Yes***				***No***	***Yes***				***No***	***Yes***	
**Predicted**	*No*	21	5		**Predicted**	*No*	19	4		**Predicted**	*No*	23	1	
	*Yes*	2	15			*Yes*	4	16			*Yes*	0	19	
**Accuracy**	**Sensitivity**	**Specificity**	**PPV**	**NPV**	**Accuracy**	**Sensitivity**	**Specificity**	**PPV**	**NPV**	**Accuracy**	**Sensitivity**	**Specificity**	**PPV**	**NPV**
0.84 (0.69–0.93)	0.91	0.75	0.81	0.88	0.81 (0.67–0.92)	0.83	0.8	0.83	0.8	*0.98 (0.88–1)*	*1*	*0.95*	*0.96*	*1*

* *In both cases, partial-least-squared discriminant analyses were run to predict the same outcome, i.e., clinical improvement after rifaximin therapy. In the first model, only sensors' responses obtained from the analysis of pre-rifaximin samples were entered as predictor, whereas, in the second model, the change in sensors' responses obtained from the analysis of both pre- and post-rifaximin samples was tested as potential predictor of clinical improvement*.

*PPV, positive predictive value; NPV, negative predictive value*.

## Discussion

In this study, we comprehensively explored the effects of rifaximin on fecal microbiota in SUDD patients, correlating them with clinical and laboratory data and assessing whether sensor-based methods can gain insight into microbiota status and its rifaximin-related changes. We showed that rifaximin significantly affects the relative abundance of selected bacteria and that an electronic multisensorial system (e-nose and e-tongue) has the potential for predicting and discriminating rifaximin-induced clinical response.

Several studies have demonstrated that rifaximin, a poorly absorbed oral antibiotic with an activity against anaerobic, gram-positive, and gram-negative bacteria, generates an “eubiotic” effect, also promoting the growth of beneficial bacteria such as *Lactobacillus* and Bifidobacteria (28, 29). Moreover, it has been reported that rifaximin decreases the metabolic activity of intestinal microbiota, increases fecal mass, and reduces bacterial overgrowth (30), and its very low absorption rates imply a low level of bacterial resistance onset (31). However, the effects of rifaximin on the intestinal microbiota are largely unknown. In fact, it seems to not affect the overall microbiome composition (32) but to induce selective depletion of a few taxa involved in the regulation of inflammation and mucosal barrier functionality (33).

In the present study, after treatment of rifaximin, an increase of Bacteroidaceae was observed in fecal microbiota of SUDD patients; even if the impact of these bacteria on SUDD pathogenesis is not known, in several studies using animal models of colitis, Bacteroidaceae or their metabolites seem to exert a protective role against inflammation (34, 35). Actually, a significant increase of *Bacteroidetes* spp., which usually represent a significant and stable part of the GI microbiota, plays an important metabolic role with the production of succinic acid, acetic acid, and in some cases propionic acid. Interestingly, propionic acid is mainly produced by the fermentation of indigested food by the microbiota in the colon, but can reach the blood compartment and the adipose tissue, where it reduces fatty acid levels in plasma *via* inhibition of lipolysis and induction of lipogenesis in adipose tissue and suppression of fatty acid production in liver (36). Moreover, after rifaximin treatment, we observed a significant reduction of Christensenellaceae, which are usually present, in a large amount, in patients with a previous history of diverticulitis, suggesting a possible role of this bacterial family in the pathogenesis of SUDD (37). Moreover, after rifaximin treatment a significant decrease of several microbial species was also observed such as *Eggerthella lenta*, which is an emerging pathogen responsible for bacteremia in several pathological conditions, among which is diverticular disease (38).

Overall, we did not observe a significant variation of serum inflammatory markers, and only less than half of the participants reported a clinical improvement after rifaximin. This could be likely due to the fact that the participants were not naïve to rifaximin. Furthermore, nearly two-thirds of enrolled subjects had normal bowel habits or absence of abdominal pain or only mild bloating at baseline. As such, the impact of rifaximin therapy in this specific cohort of not-naïve and pauci-symptomatic subjects could have been less evident.

Both e-nose and e-tongue were able to distinguish patients who ultimately will benefit from rifaximin from those who will not. However, the integration of the two methods did not improve the discrimination (prediction), as if the information inherent to (in) each technique was in itself complete and could not benefit from any integration. We remind that e-nose assesses the pattern of VOCs, while e-tongue assesses the bioelectric properties of a liquid, which reflects the chemical composition. It is noteworthy that all the components of the liquid contribute to shape its e-tongue pattern, whereas only volatile compounds contribute to the e-nose pattern. Accordingly, it is not surprising that adding information based on selected components (e-nose) to that derived from the whole set of liquid components (e-tongue) does not significantly improve the prognostic and discriminatory properties of the latter. The same consideration likely applies to the lack of improvement in the prediction of alpha diversity measures of gut microbiota by integrating e-nose and e-tongue. Unfortunately, the available literature does not provide any example of integration of e-tongue and e-nose, making our hypothesis worthy of validation in other settings. Instead, the integration of the two methods significantly improved the discrimination based on both baseline and post-rifaximin data. This seemingly contradictory (variant) finding is likely due to the distinctive effects of rifaximin on bacterial phyla. Indeed, the observed changes in loads of individual phyla might account for changing proportions of volatile compounds after rifaximin, making thus a “repeated information,” the one on volatile compounds collected by e-nose and, in their liquid form, by e-tongue, more representative of the biological changes underlying clinical changes. Finally, both e-nose and e-tongue were weakly correlated with changes in the vast majority of bacterial taxa, with only a few exceptions. Given, we found relevant changes in 7 phyla, 34 families, and 37 genera; the few significant correlations might be chance findings. However, it is of interest that they pertain to bacteria with plausible biological importance.

The present study has some limitations. First, the limited sample size makes the obtained results exploratory in nature, thus requiring confirmation in largest studies. Subgroup analyses did not reveal differences in diversity measures according to gender and to the presence of abdominal pain, and selected families and genera were found increased or decreased across strata. However, the reduced statistical power hampers sensible speculations on gender- and symptom-specific findings, and a wider cohort should be enrolled to highlight such subtle microbiological differences at the family and genus levels. Secondly, the studied cohort of SUDD subjects was not naïve to rifaximin therapy, and this might have smoothed the discriminative properties of multisensory systems. However, the fact that these properties were evident even in this “difficult” population testifies to the potentialities of the proposed method. Then, we did not assess intestinal permeability or inflammatory status, which might have helped our understanding of observed changes in microbioma. However, interpreting changes was out of the scope of our study, which was designed to test whether e-nose and e-tongue could assess rifaximin-induced changes in microbioma. Finally, the lack of concomitant stool metabolomic data prevents a more comprehensive understanding of the impact of the observed microbiota changes after therapy.

However, strengths also are worthy of mention. This is the first study characterizing the microbiota in SUDD subjects before and after rifaximin, and, as such, its results should be regarded with attention. In particular, the time by dose cumulative exposition to rifaximin chosen for treatment corresponds to the minimal one known to be clinically effective. Thus, the multisensorial system showed discriminative properties in a difficult experimental condition. It is likely that expanding the research toward stronger therapeutic regimens will further disclose the diagnostic potential of this method. Moreover, it tests a very innovative approach based on different sensor methods (electronic multisensorial system made up of e-nose and e-tongue) in the search for an easy and inexpensive method to surrogate microbiota genetic study. Finally, the e-tongue method can be easily standardized, given its dependence upon the operating electrical field, which guarantees for its reproducibility.

In conclusion, the present study highlights specific rifaximin-related changes in stool microbiota of SUDD patients in the selected bacterial population. Moreover, it shows that an electronic multisensorial system is able to efficiently predict rifaximin-induced clinical response. These preliminary results need to be confirmed and expanded in other SUDD and not SUDD populations. If confirmed, they might open the way to a fast and low-cost stool characterization with many potential perspectives of use not only in gastroenteric diseases.

## Data Availability Statement

All sequences have been archived in NCBI database: PRJNA731467 (https://www.ncbi.nlm.nih.gov/bioproject).

## Ethics Statement

The studies involving human participants were reviewed and approved by Campus Bio-Medico. The patients/participants provided their written informed consent to participate in this study.

## Author Contributions

RA, BM, MC, LP, AD, MS, and GP conceived the study. BM, MG, and AA recruited participants. AD, FD, SR, LP, and RA conducted the analysis. SG, GP, MS, and AZ analyzed breath and fecal samples. AD, RA, AA, and AL wrote the article. All authors revised and approved the written manuscript.

## Conflict of Interest

The authors declare that the research was conducted in the absence of any commercial or financial relationships that could be construed as a potential conflict of interest.
