# Supplementary material for: Gut Microbiota and Related Electronic Multisensorial System Changes in Subjects With Symptomatic Uncomplicated Diverticular Disease Undergoing Rifaximin Therapy
Source: Front Med (Lausanne). 2021 Jul 19;8:655474. doi: 10.3389/fmed.2021.655474 (PMC8326398; doi:10.3389/fmed.2021.655474)
Supplement: Supplementary file 1 [file Data_Sheet_1.docx]

**Supplementary Figure 1. Change of alfa and beta diversity measures after Rifaximin therapy in SUDD patients, stratified by gender and by presence of abdominal pain.** Alpha diversity of the gut microbiota was measured on the raw data by the Shannon and the Chao-1 index (upper panels). Beta diversity was assessed by principal component analysis (PCoA) on unweighted and weighted UniFrac distance matrix (lower panels). Wilcoxon signed rank test and permutational multivariate analysis of variance (PERMANOVA) were applied to assess differences in alfa and beta diversity measures after rifaximin therapy, respectively.

**A) Men**


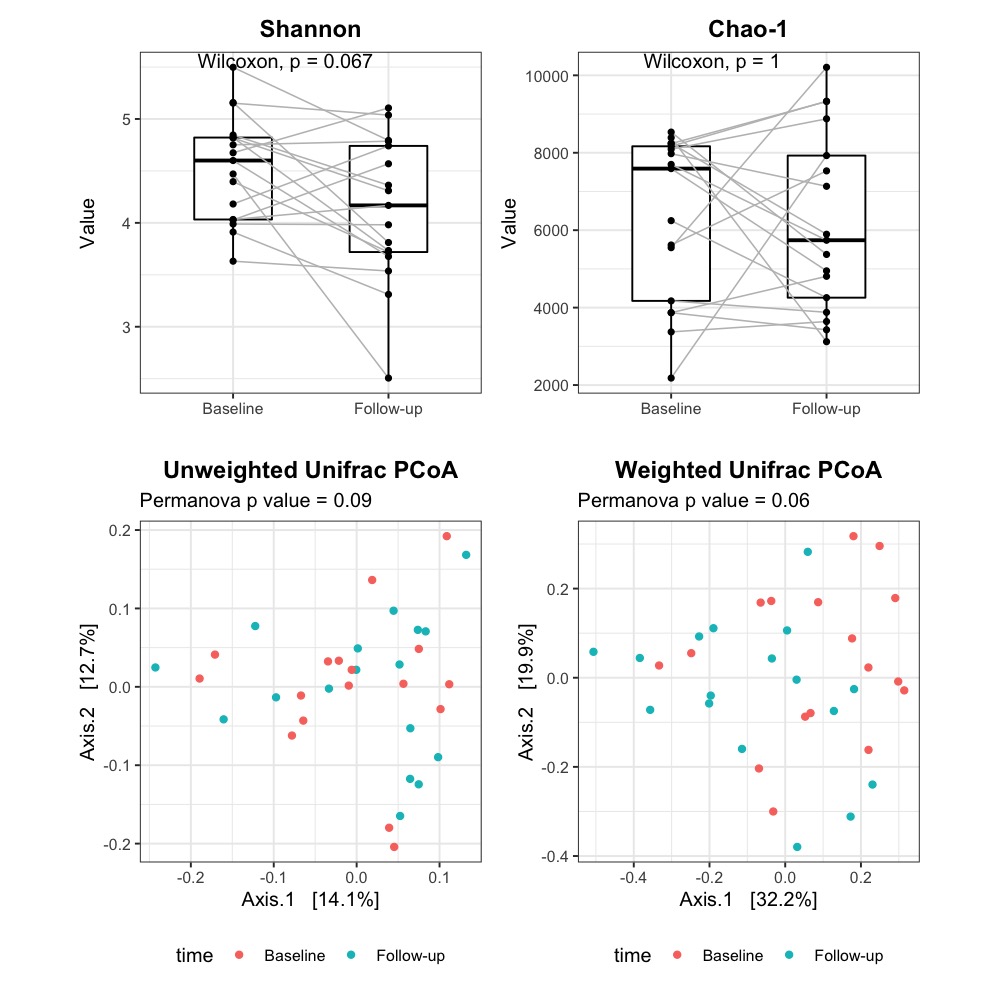


**B) Women**


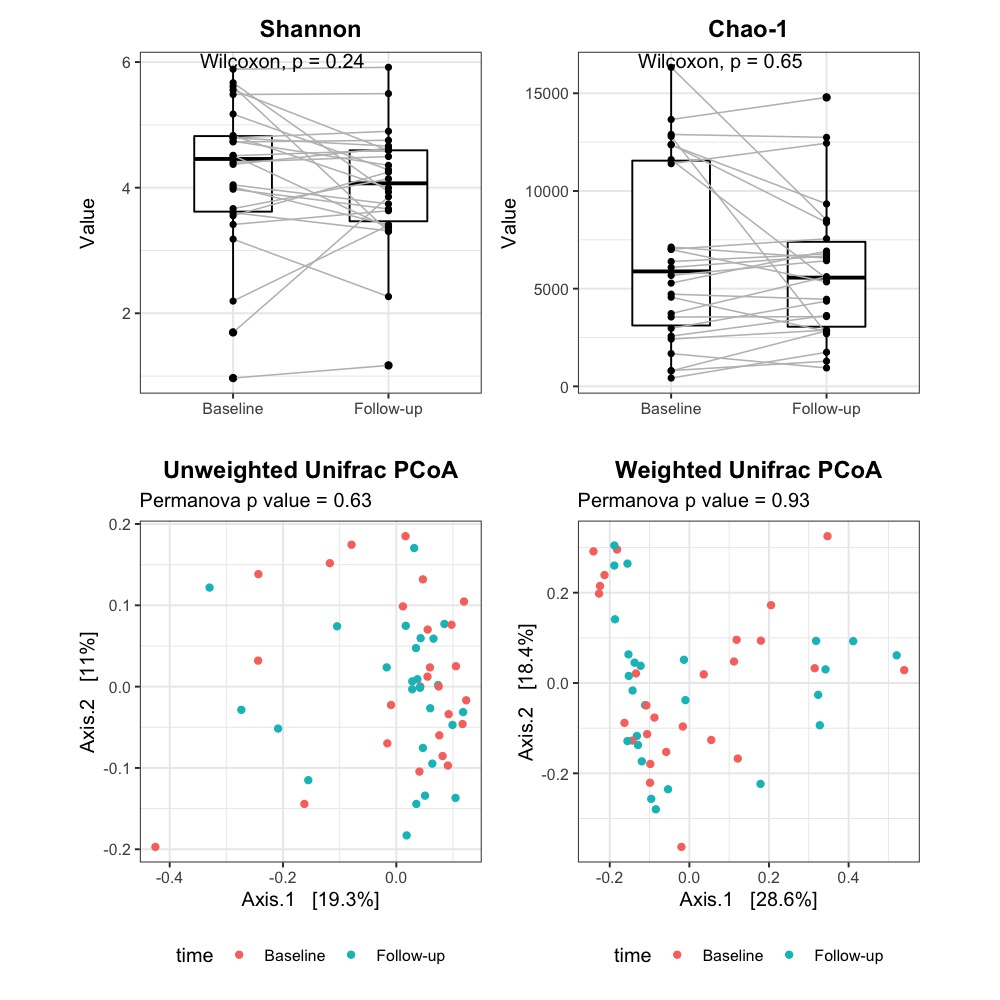


**C) Subjects without abdominal pain**


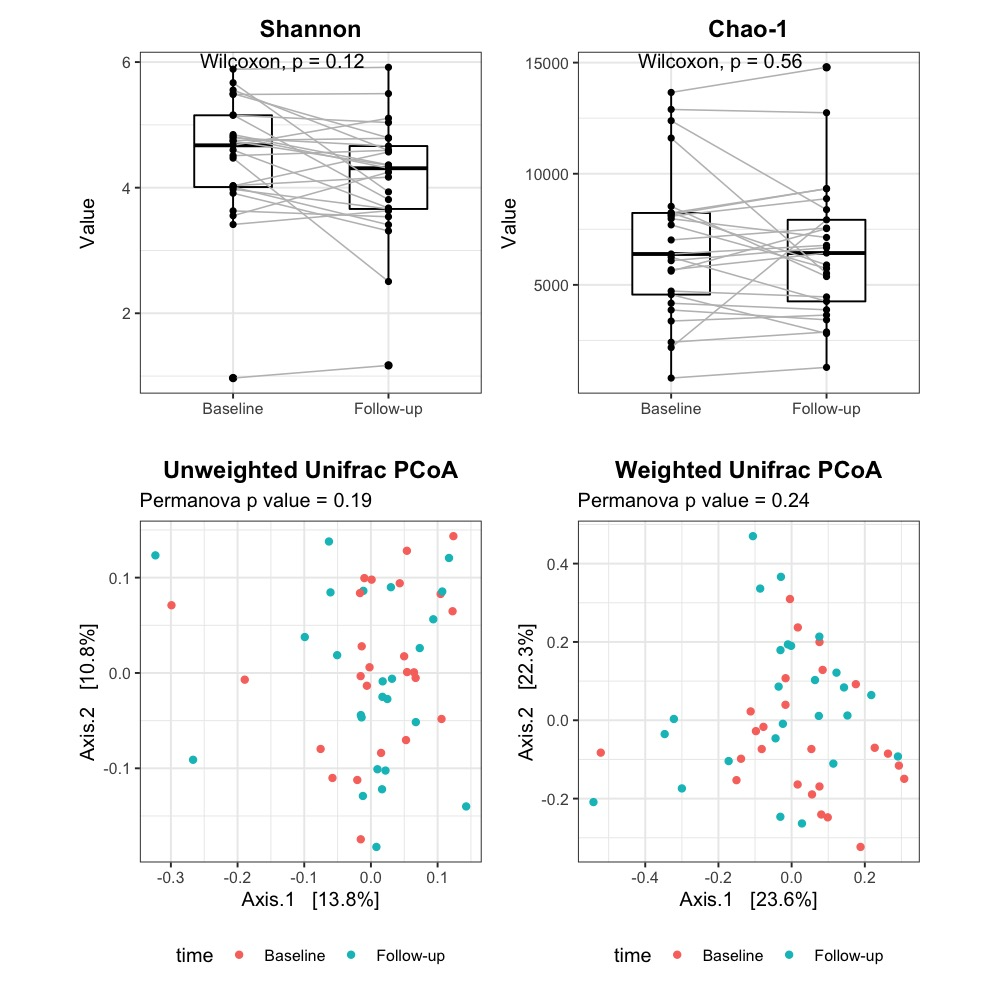


**D) Subjects with abdominal pain**


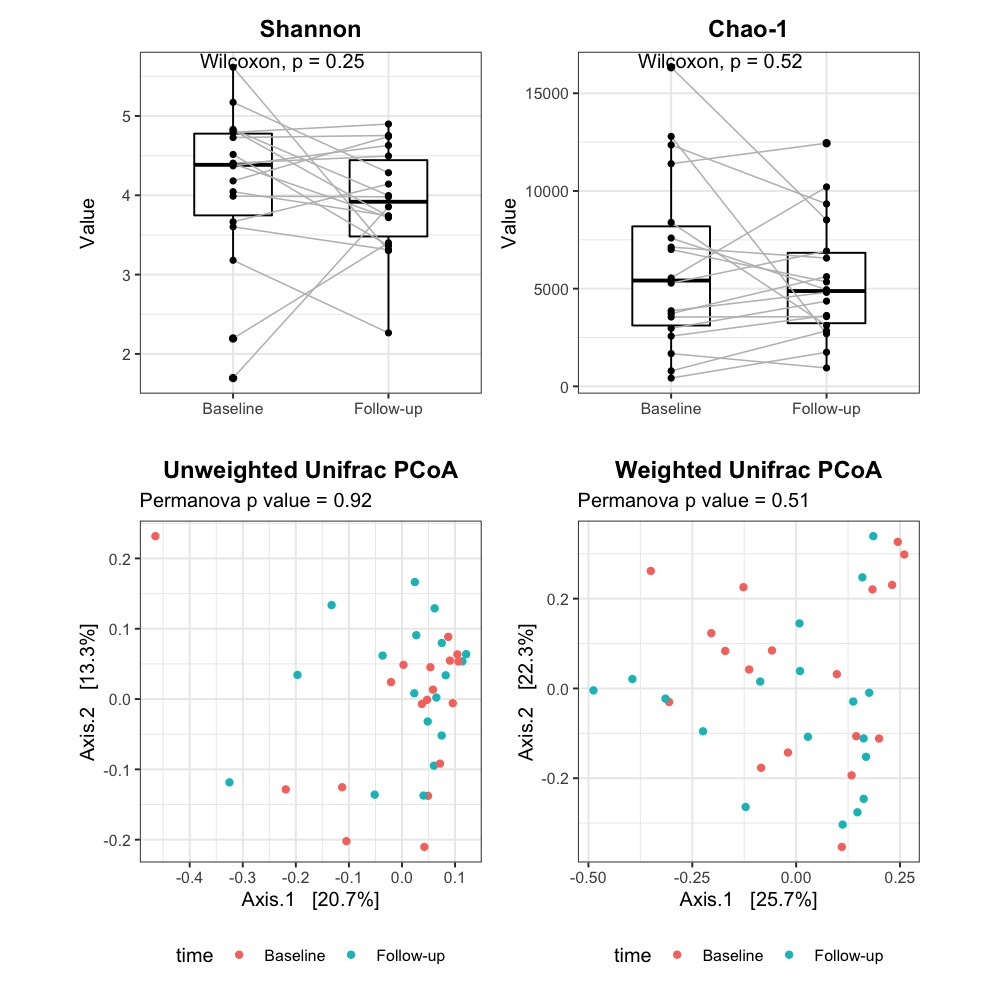


**Supplementary Figure 2. Differential abundance analysis of the gut microbiota composition at the phylum (green), family (orange) and genus (blue) level after Rifaximin therapy, stratified by gender.** Differential bacterial abundance is expressed as log2 fold change (log2FC); positive or negative values indicate an increase or decrease proportional to the absolute value of log2FC. Comparisons with a log2FC higher or lower than 0.5 are displayed. P‐value <0.05 adjusted for multiple comparisons with the Benjamini-Hochberg method (P‐adj) are considered significant and represented by a darker color.

**A) Men**


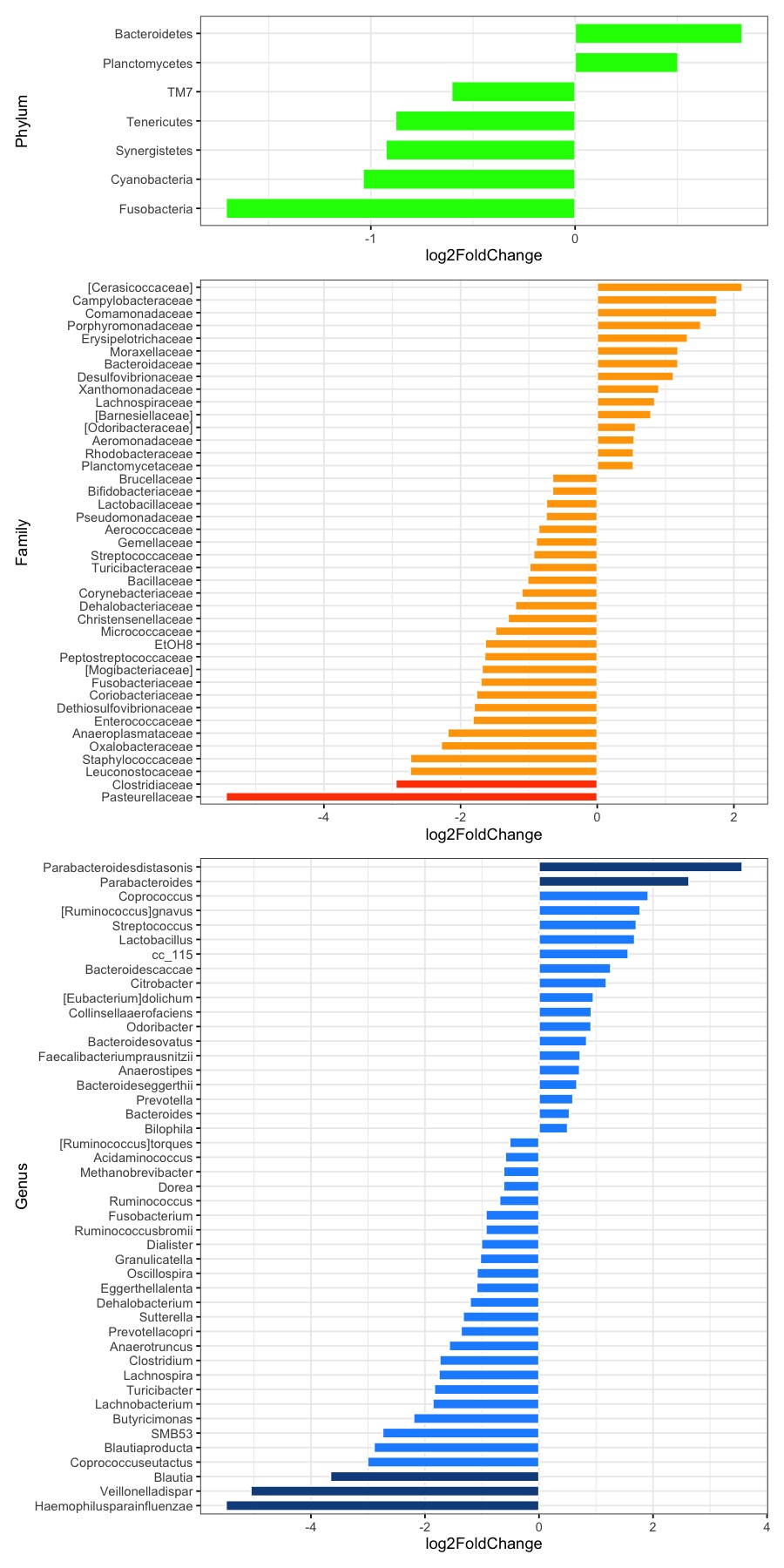


**B) Women**


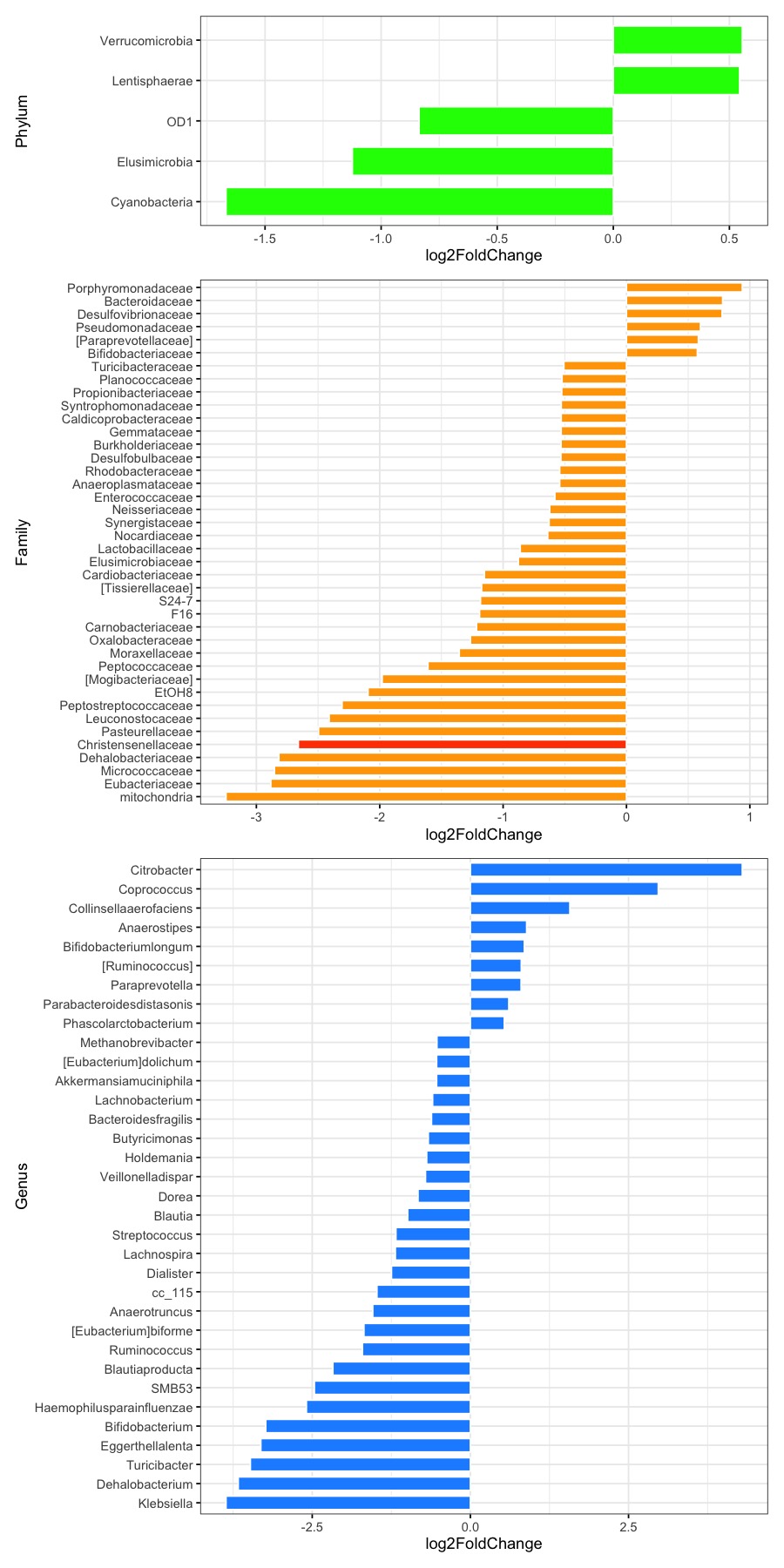


**C) Subjects without abdominal pain**


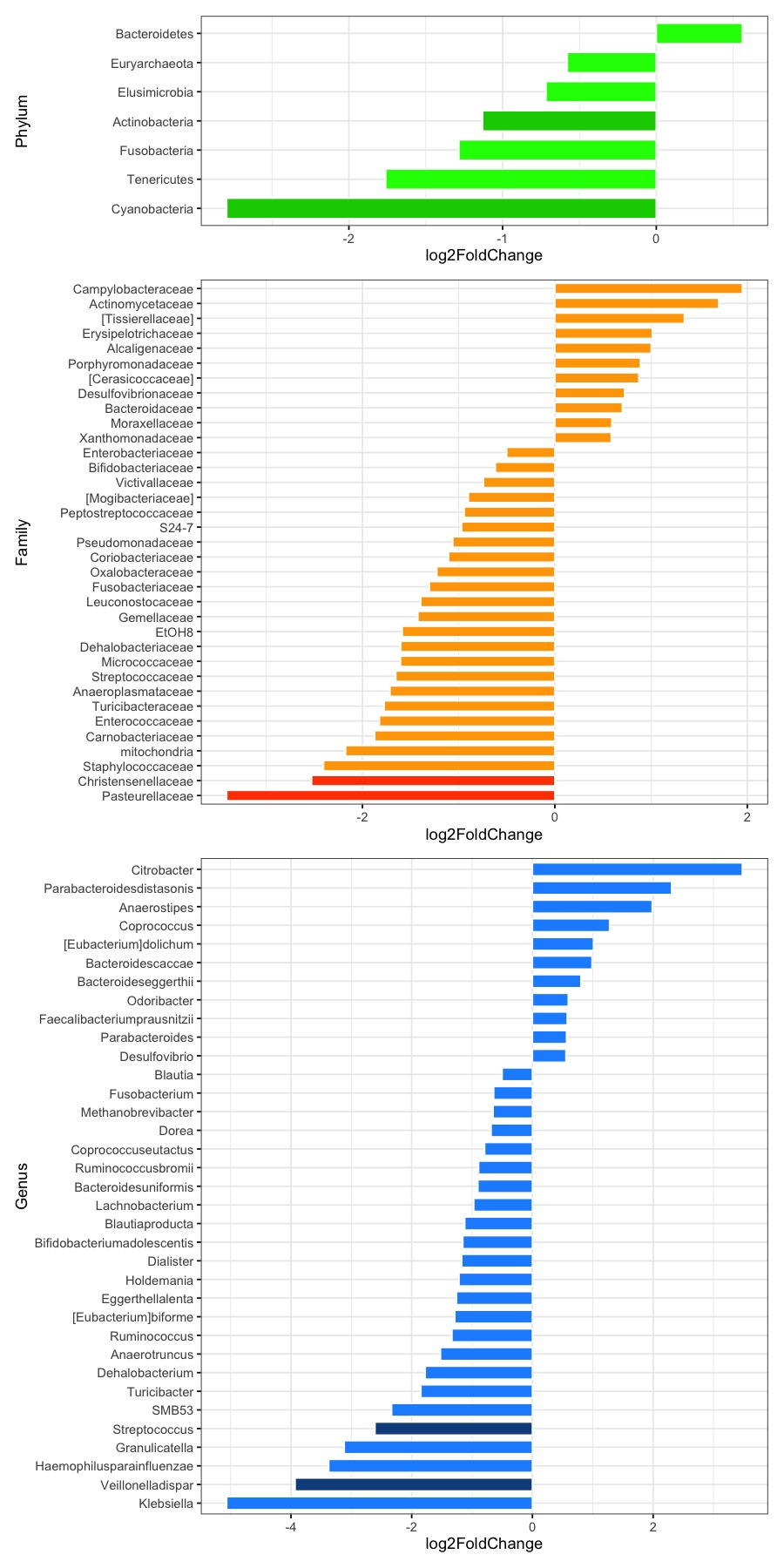


**D) Subjects with abdominal pain**


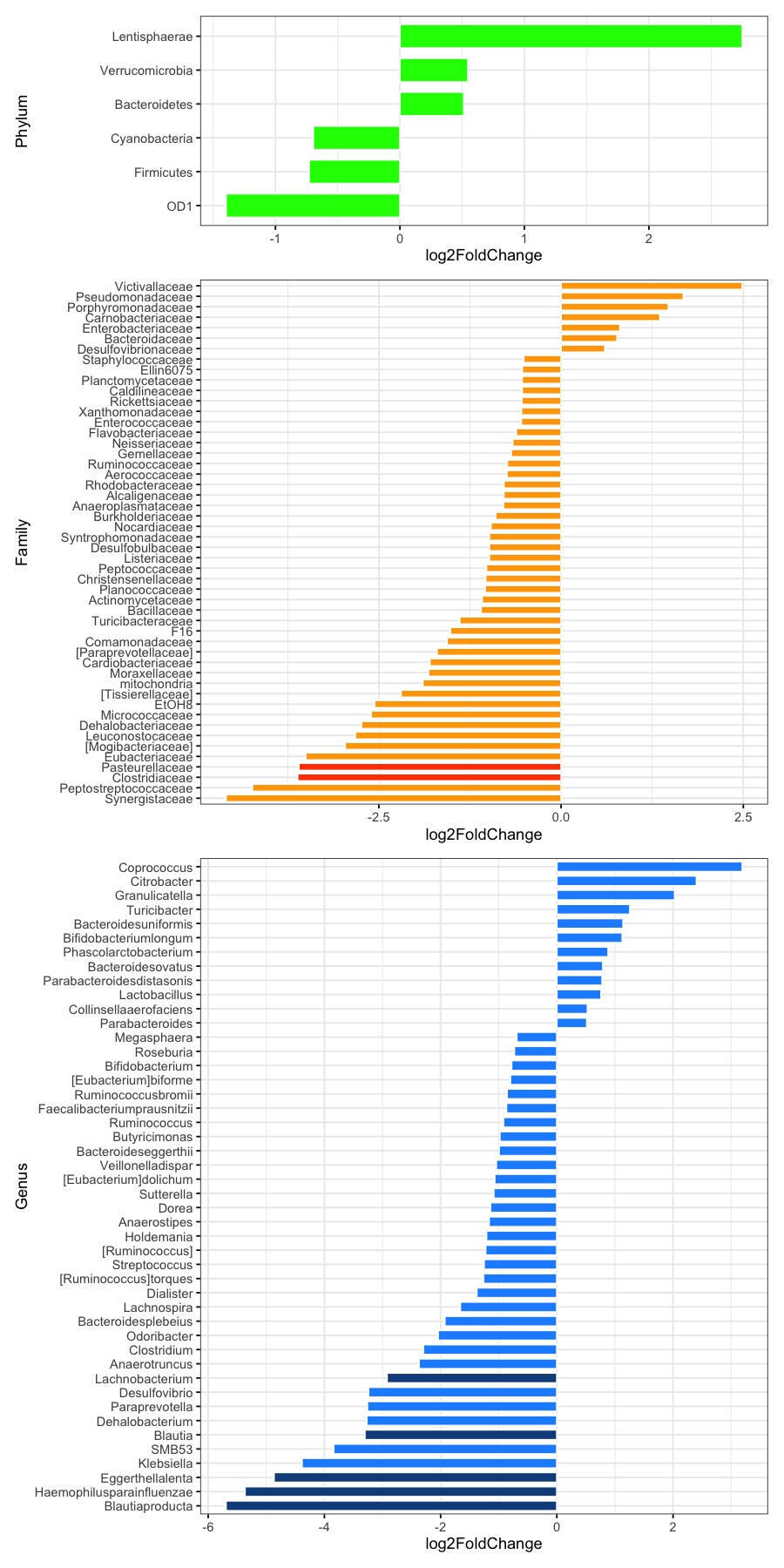


**Supplementary Table 1. E-tongue and e-nose prediction of the main phyla, families and genera variation in subjects with SUDD undergoing rifaximin therapy.**

|  | **E-TONGUE** | | **E-NOSE** | | **E-TONGUE+E-NOSE** | |
| --- | --- | --- | --- | --- | --- | --- |
|  | *RMSECV* | *RMSECV%* | *RMSECV* | *RMSECV%* | *RMSECV* | *RMSECV%* |
| **Phylum** |  |  |  |  |  |  |
| Bacteroidetes | 11295 | 20 | 13626 | 24 | ***9880*** | ***18*** |
| Verrucomicrobia | 6341 | 24 | 6384 | 25 | 6394 | 25 |
| Tenericutes | ***67*** | ***18*** | ***69*** | ***19*** | ***69*** | ***19*** |
| Fusobacteria | 968 | 265 | 935 | 256 | 967 | 265 |
| OD1 | 1 | 76 | 1 | 77 | 1 | 76 |
| Elusimicrobia | 0 | - | 0 | - | 0 | - |
| Cyanobacteria | 222 | 44 | 217 | 43 | 239 | 47 |
|  |  |  |  |  |  |  |
| **Family** |  |  |  |  |  |  |
| Bacteroidaceae | 10471 | 20 | 12090 | 23 | ***9217*** | ***17*** |
| Verrucomicrobiaceae | 6366 | 25 | 6406 | 25 | 6415 | 25 |
| Christensenellaceae | 579 | 68 | 582 | 68 | 553 | 64 |
| Clostridiaceae | 602 | 31 | 595 | 30 | 568 | 29 |
| Erysipelotrichaceae | 459 | 39 | 397 | 34 | 487 | 41 |
| [Mogibacteriaceae] | 34 | 22 | 34 | 22 | 32 | 21 |
| Dehalobacteriaceae | 16 | 27 | 17 | 29 | 17 | 28 |
| Pasteurellaceae | 142 | 35 | 144 | 36 | 138 | 34 |
| Desulfovibrionaceae | 258 | 24 | ***145*** | ***14*** | ***214*** | ***20*** |
| Fusobacteriaceae | 968 | 265 | 935 | 256 | 967 | 265 |
| mitochondria | 5 | 53 | 5 | 53 | 5 | 53 |
| Actinomycetaceae | 3 | 23 | 3 | 23 | 3 | 23 |
| Moraxellaceae | 1 | 135 | 1 | 149 | 1 | 146 |
| Anaeroplasmataceae | 3 | 263 | 1 | 104 | 3 | 272 |
| EtOH8 | 4 | 21 | 5 | 26 | 4 | 21 |
| Peptostreptococcaceae | 5 | 24 | 5 | 22 | 5 | 24 |
| Campylobacteraceae | 4 | 34 | 4 | 33 | 4 | 33 |
| F16 | 1 | 34 | 1 | 35 | 1 | 34 |
| Bacillaceae | 1 | 28 | 1 | 29 | 1 | 29 |
| Eubacteriaceae | 7 | 140 | 7 | 138 | 7 | 137 |
| Enterococcaceae | 3 | 18 | 5 | 23 | 5 | 23 |
| Leuconostocaceae | 5 | 30 | 7 | 37 | 7 | 37 |
| Cardiobacteriaceae | 0 | 25 | 0 | 25 | 0 | 25 |
| Peptococcaceae | 9 | 30 | 9 | 31 | 9 | 29 |
| Micrococcaceae | 5 | 25 | 5 | 26 | 5 | 28 |
| S24-7 | 672 | 46 | 708 | 49 | 686 | 47 |
| Carnobacteriaceae | 12 | 38 | 12 | 39 | 12 | 39 |
| Staphylococcaceae | 7 | 350 | 7 | 354 | 7 | 355 |
| Turicibacteraceae | 90 | 39 | 95 | 41 | 96 | 42 |
| Porphyromonadaceae | 1331 | 24 | 1160 | 21 | 1422 | 25 |
| Elusimicrobiaceae | 0 | Inf | 0 | Inf | 0 | Inf |
| Gemellaceae | 2 | 23 | 2 | 25 | 2 | 25 |
| Oxalobacteraceae | 12 | 34 | 15 | 44 | 13 | 38 |
| Aerococcaceae | 4 | 81 | 4 | 82 | 4 | 82 |
|  |  |  |  |  |  |  |
| **Genus** |  |  |  |  |  |  |
| Dialister | ***1837*** | ***20*** | 1950 | 22 | 1950 | 22 |
| Lachnospira | 1644 | 25 | 1626 | 25 | 1626 | 25 |
| Oscillospira | 482 | 38 | 725 | 57 | 725 | 57 |
| Ruminococcus | 631 | 22 | 626 | 21 | 626 | 21 |
| Ruminococcusbromii | 1251 | 30 | 1312 | 31 | 1312 | 31 |
| Odoribacter | 112 | 34 | 110 | 33 | 110 | 33 |
| Roseburia | 824 | 35 | 723 | 31 | 723 | 31 |
| Coprococcuseutactus | 78 | 30 | 81 | 32 | 81 | 32 |
| Bifidobacteriumlongum | 347 | 87 | 369 | 93 | 369 | 93 |
| Holdemania | 37 | 22 | ***31*** | ***19*** | ***31*** | ***19*** |
| Bilophila | 55 | 21 | 54 | 21 | 54 | 21 |
| Streptococcus | 193 | 46 | 194 | 46 | 194 | 46 |
| Dehalobacterium | 6 | 34 | 6 | 36 | 6 | 36 |
| Haemophilusparainfluenzae | 121 | 33 | 123 | 34 | 123 | 34 |
| Blautia | 423 | 36 | 399 | 34 | 399 | 34 |
| Lachnobacterium | 406 | 27 | 403 | 27 | 403 | 27 |
| Dorea | 112 | 24 | 114 | 25 | 114 | 25 |
| Veillonelladispar | 2150 | 42 | 2030 | 40 | 2030 | 40 |
| Klebsiella | 36 | 33 | 36 | 33 | 36 | 33 |
| Eggerthellalenta | 16 | 25 | 19 | 30 | 19 | 30 |
| Bacteroidesuniformis | ***3003*** | ***16*** | ***3356*** | ***18*** | ***3356*** | ***18*** |
| Sutterella | 619 | 46 | 819 | 60 | 819 | 60 |
| Citrobacter | 16 | 23 | 16 | 23 | 16 | 23 |
| Anaerotruncus | 31 | 59 | 30 | 57 | 30 | 57 |
| [Eubacterium]biforme | 57 | 111 | 53 | 104 | 53 | 104 |
| Bifidobacterium | 54 | 561 | 50 | 518 | 50 | 518 |
| Bacteroidescaccae | 45 | 32 | 45 | 33 | 45 | 33 |
| SMB53 | 19 | 26 | 20 | 28 | 20 | 28 |
| Parabacteroidesdistasonis | 53 | 27 | 53 | 27 | 53 | 27 |
| Methanobrevibacter | 188 | 37 | 176 | 35 | 176 | 35 |
| Clostridium | 140 | 184 | 135 | 177 | 135 | 177 |
| Coprococcus | 23 | 28 | 29 | 35 | 29 | 35 |
| Butyricimonas | 67 | 21 | 70 | 22 | 70 | 22 |
| Granulicatella | 11 | 39 | 12 | 40 | 12 | 40 |
| Blautiaproducta | 12 | 29 | 13 | 31 | 13 | 31 |
| Turicibacter | 87 | 42 | 92 | 45 | 92 | 45 |
| [Ruminococcus] | ***11*** | ***16*** | ***13*** | ***19*** | ***13*** | ***19*** |

RMSECV% represents the ratio between the root mean squared error in cross validation (RMSECV) and the 95% interval of the distribution of values for each phylum, family and genus change pre-post rifaximin therapy. Only phyla, families and genera that showed a log2 fold change higher or lower than 0.5 have been analysed. Predictions with a RMSECV below 20% have been highlighted in **bold**.
